# Challenges in Using Circulating miRNAs as Cancer Biomarkers

**DOI:** 10.1155/2015/731479

**Published:** 2015-03-22

**Authors:** Paola Tiberio, Maurizio Callari, Valentina Angeloni, Maria Grazia Daidone, Valentina Appierto

**Affiliations:** Biomarker Unit, Department of Experimental Oncology and Molecular Medicine, Fondazione IRCCS Istituto Nazionale dei Tumori, Via Amadeo 42, 20133 Milan, Italy

## Abstract

In the last years, circulating miRNAs have emerged as a new class of promising cancer biomarkers. Independent studies have shown the feasibility of using these small RNAs as tools for the diagnosis and prognosis of different types of malignancies as well as for predicting and possibly monitoring treatment response. However, despite an initial enthusiasm for their possible clinical application, widespread inconsistencies have been observed among the studies, and miRNA-based tools still represent the object of research within clinical diagnostic or treatment protocols. The poor overlap of results could be explained, at least in part, by preanalytical and analytical variables and donor-related factors that could generate artefacts, impairing an accurate quantification of circulating miRNAs. In fact, critical issues are represented by nonuniform sample choice, handling, and processing, as well as by blood cell contamination in sample preparation and lack of consensus for data normalization. In this review, we address the potential technical biases and individual-related parameters that can influence circulating miRNA studies' outcome. The exciting potential of circulating miRNAs as cancer biomarkers could confer an important advance in the disease management, but their clinical significance might not be proven without a global consensus of procedures and standardized protocols for their accurate detection.

## 1. Introduction

MicroRNAs (miRNAs) are highly conserved single-stranded small RNA molecules (~19–22 nucleotides long) that play a key role in posttranscriptional gene regulation [1, 2]. To date, more than 2,500 human miRNAs have been identified (miRBase V20, [3]). These small RNA molecules bind the 3′UTR region of their messenger RNA (mRNA) targets, inducing posttranscriptional gene regulation by either inhibition of translation or mRNA degradation [2, 4, 5], and are being widely investigated with multiple approaches in oncology [6]. Indeed, in the last decade their implication in different types of cancer has become clear [7], generating a great deal of enthusiasm in clinical and scientific communities. miRNA signatures from normal cancer tissues and metastases have been used to classify different types of cancer and have been shown to represent potential biomarkers for diagnosis, prognosis, and therapy [8, 9]. In addition, recent studies have shown that miRNAs can be released from cells (encapsulated in exosomes and/or bound to proteins and lipoproteins) and enter the circulation as a consequence of apoptotic and necrotic cell death, as well as of an active release [10–15]. As a result of miRNA release from cells, these molecules have been also found in several human body fluids (including blood, serum, plasma, urine, saliva, seminal fluid, and pleural effusion) [16], in a stable form protected from endogenous RNAses, thus making circulating miRNA levels well suited for noninvasive analysis in patient samples [17–19]. Independent studies have reported the feasibility of using circulating miRNAs as promising disease biomarkers and, in the context of malignancies, they have shown a potential as molecular tools for detection, prognosis, and treatment decision making of various cancers [17–23]. In this review, we focus on studies and technical issues concerning the evaluation of circulating miRNAs in blood, plasma, and serum, the biological fluids most analyzed as a source of cell-free miRNAs.

## 2. Circulating miRNAs as Promising Cancer Biomarkers

In the last few years, there has been increasing interest in circulating miRNAs as cancer biomarkers, due to their high stability, their putative capability to be more informative than mRNA, and the noninvasiveness of their detection. Since their discovery in body fluids, considerable effort has been directed to investigate the relevance of these small RNAs in different diseases, and now there is much evidence of their potential clinical relevance as cancer biomarkers in different types of malignancies. The first study that identified specific circulating miRNAs associated to cancer was by Lawrie et al. [24], who in 2008 found high levels of miR-155, miR-210, and miR-21 in patients with diffuse large B-cell lymphoma and demonstrated a significant correlation between high levels of miR-21 and relapse-free survival. A literature survey concerning the principal findings related to the usefulness of specific circulating miRNAs (or miRNA signatures) as diagnostic, prognostic, and/or predictive parameters in different cancer entities has recently been published (reviewed in [23]). Plasma or serum miRNAs appear to display a promising potential mainly in the diagnosis of different solid tumors at preoperative level, thus suggesting the possibility of their utility as early-diagnostic tools. Accordingly, a very recent study performed in a large cohort of smoker individuals provided evidence that specific ratio-based miRNA signatures (including 24 distinct miRNAs assayed in plasma samples) have significant diagnostic and prognostic power to anticipate the detection of malignant lung cancers and to predict tumor aggressiveness [25].

It should be pointed out that circulating miRNAs might not only represent promising noninvasive diagnostic and prognostic tools but they could also be used to predict and monitor the efficacy of anticancer treatments. In this context, recent correlative studies within neoadjuvant or adjuvant chemotherapy trials identified many circulating miRNAs as associated with response to treatment and drug resistance [23]. For example, in HER2-positive breast cancer patients undergoing neoadjuvant therapy, plasma miR-210 levels were found to be associated to trastuzumab sensitivity, thus suggesting that plasma miR-210 levels might be used to predict and monitor response to therapies containing the monoclonal antibody [26]. In the context of adjuvant chemotherapy regimens, it has been shown that serum miR-21 levels can predict the benefit of gemcitabine treatment in advanced pancreatic cancer patients, suggesting that the miRNA might be used as a predictor of the chemosensitivity to this nucleoside analogue [27]. However, despite the fact that several published papers demonstrated the feasibility of using circulating miRNAs as putative cancer biomarkers, many preanalytical and analytical aspects, as well as donor-related factors, can interfere with accurate circulating miRNA quantification, and future studies have to take them into consideration.

## 3. Preanalytical and Analytical Variables Affecting Circulating miRNA Studies

**Figure 1:**
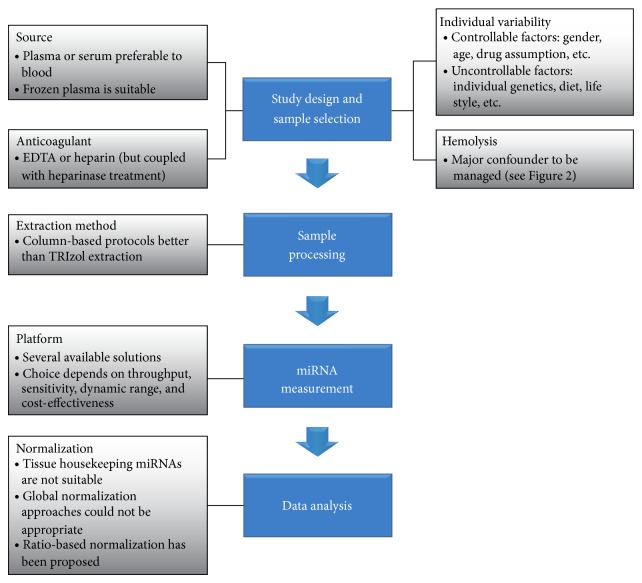
Flow chart summarizing the different steps and the main preanalytical and analytical confounders in circulating miRNA detection.

In a recent work by Leidner et al. [28], the authors highlighted for the first time the widespread inconsistency across circulating miRNA studies, cautioning the scientific community about the huge variety of methodological parameters impairing circulating miRNA evaluation.  Figure 1 summarizes the main preanalytical and analytical variables interfering with circulating miRNA analysis that are examined in detail in the following sections.

### 3.1. Sample Choice/Starting Material

Quantification of miRNAs present in the circulation can be performed from different sources of materials (i.e., whole blood, plasma, and serum). In a recent study, Pritchard et al. [29] demonstrated the influence of blood cell miRNAs (contained in red and white blood cells and in platelets) in circulating miRNA analysis. They verified that blood cells are substantial contributors to the presence of miRNAs in the circulation, markedly altering specific miRNA levels. For this reason, whole blood may not be considered a preferential biological fluid for circulating miRNA detection. However, even for the studies performed in plasma (liquid part of the blood containing fibrinogen and collected in the presence of an anticoagulant) and serum (liquid part of the blood obtained after the blood has clotted), the complete removal of cellular components that could impair accurate miRNA quantification is mandatory. Many research groups have compared different plasma/serum preparation protocols, analyzing differences between the two types of biological fluid in terms of circulating miRNA amount, but univocal results have not been obtained. In fact, McDonald et al. [30] reported that plasma presents higher level of specific miRNAs than does serum, which is in contrast to results shown by Mitchell et al. [17] and by Wang et al. [31]. To explain this difference, the latter group has hypothesized that the higher miRNA concentration observed in serum compared to plasma may be due to miRNA release from blood cells (such as platelets) during the coagulation process [31]. In addition, the inconsistency between the two body fluids could also be explained by the different methods used for the separation of plasma and serum from whole blood, which could lead to different amounts of blood cell contamination in these fluids. In fact, it is known that different centrifugation protocols can produce platelet-rich or platelet-poor plasma [32, 33]. Specifically, considering plasma preparation protocols, many research groups recommend a double step of centrifugation (even though slight differences regarding centrifugation speed and time can be found in the different protocols) for plasma separation in order to limit platelet contamination [33–35]. In addition, the choice of the anticoagulant used to collect blood for plasma preparation could impair further analysis. For many years, the use of heparin in blood collection tubes dedicated to RNA analysis has been avoided since the anticoagulant inhibits PCR amplification [32, 36–38]. However, we have recently demonstrated that if adequately treated (i.e., performing digestion with heparinase on extracted RNA), plasma samples derived from blood collected with heparin tubes may be suitable for miRNA expression analysis, without affecting miRNA detection performance [39]. EDTA and citrate, two anticoagulants commonly added to blood collection tubes, are suitable for miRNA detection without any additional treatment [38]. However, EDTA collection tubes should be preferred to citrate because the latter may trigger hemolysis (see the following section about hemolysis interference on miRNA analysis) [40]. Finally, interesting findings have been obtained comparing fresh and frozen fluids. In accordance with the fact that miRNAs are highly stable in the circulation, minimal or no differences have been found between fresh and frozen specimens, even after repeated freeze-thaw cycles [17, 33, 41–43]. Nevertheless, it is advisable to avoid any unnecessary freeze-thawing, since, if miRNA degradation occurs (even to a limited extent), poorly represented miRNA species could be missed. Other critical concerns regarding the starting material have been raised. For example, since skin cells (containing tissue-specific miRNAs) could also contaminate the first blood draw [32], it would be more advisable to discard it. Moreover, the fact that plasma/serum samples from patients with an inflammatory process could be contaminated with a high number of white blood cells should be taken into account [32]. Exclusion of such patients from miRNA analysis could thus be advisable. In this section the technical issues known to be strictly associated with blood collection and serum/plasma preparation have been examined, but we cannot exclude a putative impact of other unknown factors on miRNA profiling. Thus, to minimize confounding effects, at least specific standard operating procedures (SOPs) for blood collection and plasma/serum preparation are needed.

### 3.2. Hemolysis Interference

It has been demonstrated that quantification of plasma/serum miRNAs can be impaired by the contamination of erythrocyte-specific miRNAs [30, 44, 45]. Such findings have important implications for the interpretation of circulating miRNA profile results. In fact, a recent study showed that, among tumor-associated circulating miRNAs reported in the literature, 58% were highly expressed in blood cells and that hemolysis alters circulating miRNA levels by up to 50-fold [29], starting to have an influence from an erythrocyte contamination as little as 0.008% [44]. To limit hemolysis impact, the main objective would be to prevent the phenomenon.* In vivo* hemolysis cannot be avoided, but by following a few guidelines together with the definition of SOPs,* in vitro* hemolysis could be dramatically reduced. Since it has been widely proven that the mere visual detection of samples (pink or red coloration) is not sufficiently sensitive in this context, the identification of hemolyzed samples remains a crucial issue for biomarker research [46]. In the field of circulating biomarker and miRNA research studies, many approaches have been applied to identify hemolyzed plasma/serum specimens. Spectrophotometric measurement of the main oxyhemoglobin peak absorbance at wavelength (*λ*) = 414 nm could in theory represent a simple way to identify hemolyzed samples [44]. However, measuring absorbance at *λ* = 414 nm cannot be used as a robust method to identify hemolyzed samples, since, for example, lipemia in plasma interferes with hemoglobin absorbance, causing an increase in absorbance values at *λ* = 414 even in the absence of any hemoglobin-related peak. We have recently proposed a simple, sensitive, and rapid to use, lipemia-independent, spectrophotometrically based procedure able to identify, as hemolyzed, those samples containing at least 6.1 mg/dL of free hemoglobin. The preanalytical method can be performed using a NanoDrop spectrophotometer (which requires a small volume for analysis) and is based on absorbance measurements at *λ* = 414 nm and at *λ* = 385 nm (as a lipemia indicator) [47]. Otherwise, alternative methods to identify hemolyzed specimens based on the detection of erythrocyte-specific miRNAs have been recently proposed [35, 48]. Such approaches are sustained by the recent finding that a very low percentage of hemolysis can elicit a considerable increase in erythrocyte-specific miRNA levels [44, 45]. Therefore, erythrocyte-specific-miRNA-based methods are in theory highly sensitive to identify hemolyzed samples. However, contrary to preanalytical methods, miRNA quantification requires sample processing (RNA extraction) and analysis (e.g., Real-Time PCR), which is sample and time consuming. Thus, miRNA-based procedures may not be suitable if a limited amount of plasma/serum specimens is available. In addition, circulating miRNA expression can be affected by several factors, including individual variability and medical conditions (see the following section). Therefore, miRNA-based methods could turn out to be not sufficiently accurate to discriminate hemolyzed samples from samples presenting altered erythrocyte-contained miRNA expression due to other conditions.

**Figure 2:**
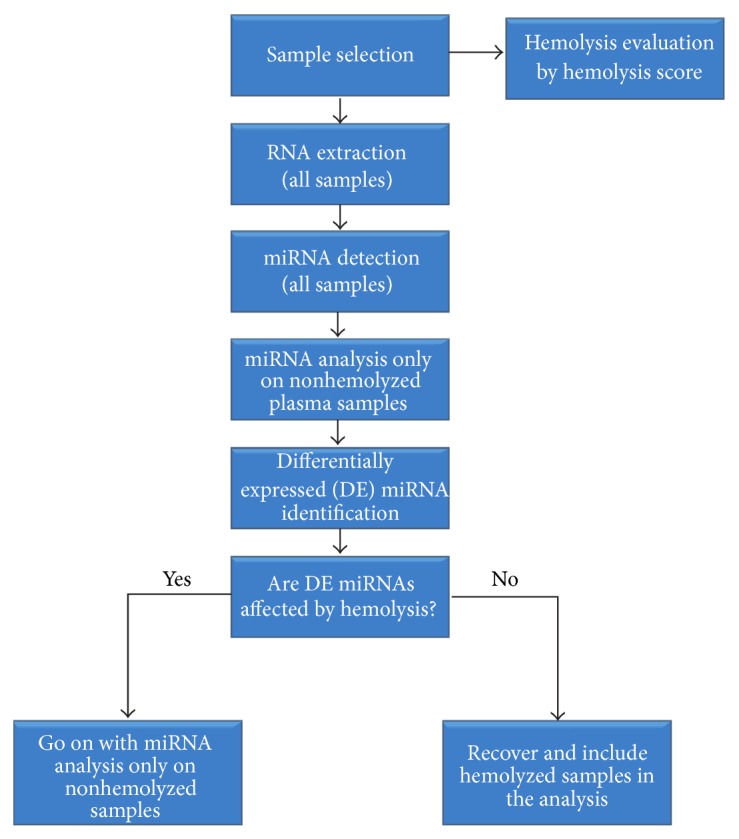
Proposed workflow for circulating miRNA studies.

Once hemolyzed samples are identified (independently of the approach used), the choice of removing them from miRNA analysis could be questionable. In fact, in our opinion, circulating miRNA studies should be performed following the workflow proposed in Figure 2. Briefly, after hemolysis evaluation, all samples have to be subjected to miRNA detection, but only nonhemolyzed samples have to be initially used for marker discovery. Then, when interesting miRNAs have been identified, researchers should determine whether (and in what extent) the identified miRNAs are hemolysis affected. If identified miRNAs are not influenced by hemolysis, hemolyzed samples could be recovered and included in the analysis.

### 3.3. Extraction Methods

Several extraction methods have been used in circulating miRNA studies. They could be divided into two different main categories: guanidine/phenol/chloroform-based protocols and commercial kits using columns or beads. Several research groups have investigated the miRNA extraction issue by comparing different protocols. First of all, McDonald et al. [30] measured four plasma/serum miRNAs to be able to compare four different extraction methods. They found that mirVana PARIS kit (Life Technologies) and miRNeasy Mini Kit (Qiagen) had the highest mean yield for miR-15b and miR-16 and for mi-24 and cel-miR-39, respectively. Similar findings have been obtained by Sourvinou et al. [49], who demonstrated that the mirVana PARIS kit and miRNeasy Mini Kit produced the highest yield of recovery for a spike-in miRNA compared to TRIzol extraction, with the first kit also obtaining a better performance than miRNeasy. However, Survinou's results were in contrast with those obtained by Kroh et al. [32] demonstrating that RNA extraction with miRNeasy led to a 2- to 3-fold increase in RNA yield compared to mirVana. More recently, Moret et al. [34] compared four different protocols for RNA extraction (using TRIzol-LS, mirVana PARIS kit, miRNeasy Serum/Plasma kit, and TRIzol-LS following mirVana kit) starting from both fresh and frozen plasma samples. The authors demonstrated that column-based methods performed better than TRIzol extraction, due to the presence of organic and phenolic contaminants in the TRIzol-extracted RNA. By comparing commercial column-based kits, they found that the miRNeasy kit obtained the highest RNA concentration and that the addition of a RNA carrier to the lysis buffer improved RNA recovery. According to such findings, Page et al. [42] compared three different commercial kits for RNA extraction and demonstrated that the miRNeasy Serum/Plasma kit produced the highest recovery of circulating RNA as regards quantity (in terms of amount of total RNA and evaluated as threshold cycle (CT) for individual miRNAs) and quality (addressed as RNA integrity). Of note, the authors also suggested that increasing starting material volume caused a less efficient recovery of miRNAs. More recently, another commercial kit has been developed for biofluid-specific RNA extraction (named miRCURY biofluids kit, Exiqon). In the only report comparing RNA extraction performed by this commercial kit with other RNA isolation methods, the authors found that the Exiqon biofluid-specific kit outperformed both Exiqon miRCURY cell and plant and Qiagen miRNeasy Serum/Plasma kits [50]. Notably, another benefit of this extraction method is the lack of a phase-separation step, thus leading to short processing time and ease of use. Despite the dissimilar results obtained in different studies, a common warning emerged about the use of TRIzol to extract miRNAs from body fluids. Conversely, no consistent results have been obtained regarding differences in column-based methods, suggesting that a great effort is still needed in comparing different extraction methods and working toward standardization.

Another warning that emerged from the studies regards plasma RNA quantification. Many research groups, independently of the specific miRNA detection methods used, have performed plasma RNA quantification by NanoDrop spectrophotometer before analyzing it for miRNA contents. However, RNA extracted from plasma/serum is undetectable by using NanoDrop spectrophotometer [30, 32, 34]. In addition, it should be considered that many diseases, including cancer, may cause a release of nucleic acids in the circulation, leading to a significant higher level of circulating RNA in cancer patients than in healthy subjects [51]. Thus, for circulating biomarker detection analysis, it could be more accurate to use an equal volume input rather than the same amount of RNA [52].

### 3.4. Detection Platforms

The accurate measurement of miRNAs, both in tissues and in circulation, poses several challenges related to their short lengths, their GC content, and the high sequence similarity within miRNA families [29]. In circulating miRNA quantification, the low abundance of these nucleic acids in body fluids has to be added to such concerns. Nonetheless, several technologies able to measure from one to thousands of miRNAs have been applied to the study of circulating miRNAs. Here we focus on three approaches that allow a medium/high throughput: amplification-based methods, hybridization-based miRNA microarrays, and massively parallel miRNA sequencing (miRNA-seq).

*Amplification-Based Methods*. Quantitative reverse transcription PCR (qRT-PCR) is a well-established method, considered as the “gold standard” for miRNA detection and is often intended as a single miRNA assay. However, assays enabling the measurement of a panel of miRNAs have been developed, such as microfluidic cards and arrays (e.g., TaqMan OpenArray and TaqMan TLDA microfluidic cards by Applied Biosystems, miRCURY LNA qPCR by Exiqon, miScript miRNA PCR Array by Qiagen). Despite the differences in the type of technology offered, all these panels allow the detection of about 700 miRNAs with high sensitivity, high specificity (single nucleotide discrimination), and high dynamic range, starting from less than 100 ng of RNA input. In addition, the data generated from this type of analysis are in terms of CT and, thus, do not necessitate further bioinformatics manipulation. However, such technology also presents some disadvantages, such as the possibility to detect only annotated miRNAs and the fact that only a medium throughput can be reached [23, 29, 52, 53]. Finally, it should be pointed out that a comparison between the two most commonly used qRT-PCR arrays (i.e., TLDA card and miRCURY array) demonstrated that both plates exhibit high reproducibility between technical replicates and that there was a significant correlation between results obtained with the two platforms [42].

*Hybridization-Based miRNA Microarrays*. Several commercial miRNA microarray platforms (e.g., GeneChip miRNA Array by Affimetrix and Human miRNA Microarray by Agilent) are available and can be used to measure circulating miRNAs. They are able to analyze up to thousands of miRNAs in one assay, but only among those already known and annotated in miRBase [3], starting from about 100 ng of total RNA. The methodology is high-throughput and is less expensive than amplification-based arrays, but it is typically considered to have lower dynamic range and specificity than qRT-PCR and miRNA-seq [23, 29, 52, 53]. Some cross-platform studies performed by our group and others [54, 55] have already highlighted limitations in correlation between results obtained with different systems for tissue miRNAs. The Agilent system emerged as one of those obtaining the highest performances and is probably the most commonly used. In a pilot study that we recently published, the feasibility of such a platform in miRNA detection also from archival plasma samples was evaluated [56]. We found a very high correlation between technical replicates and a good correlation between different batches. However, we also noticed that since circulating miRNAs give lower signals than do tissue miRNAs, the increase in chip background, when approaching the expiration date, dramatically reduced the number of miRNAs that can be detected [56].

*miRNA-Seq*. miRNA quantification by miRNA-seq is an expanding approach useful especially for miRNA families differing for a single nucleotide as well as for isomiRs of varying length. It has the great advantage of allowing detection of both known and novel miRNAs, since only a portion of the miRNA population is presently* bona fide* annotated on miRBase. Standard protocols require a relatively large amount of starting material (~1 *μ*g of RNA), which is hard to isolate from serum or plasma. However, adapted protocols have been recently proposed, giving the opportunity to obtain miRNA-seq data from as little as 5 ng of RNA extracted from plasma samples [57]. It is plausible to speculate that, in the near future, nucleic acid researches will be probably all performed using this methodology. However, for now, this technology is expensive and necessitates special equipment and expert bioinformaticians, so it cannot be considered as user and lab friendly.

### 3.5. Normalization and Data Analysis

Once we have obtained the data, the next challenge is normalization. As is known, technical variability among samples is expected due to issues such as variation in the starting material, RNA extraction, or reaction efficiency during labeling procedures or hybridization.

Housekeeping transcripts used for tissue miRNA analysis (e.g., RNU6 and RNU48) cannot be consistently detected in the circulation for their extensive RNAse-mediated degradation [31]. miR-16 is among the proposed miRNA housekeeping and probably is the most commonly reported in the literature. However, it is one of the most affected by hemolysis [29, 44] and thus may not be considered a preferential miRNA reference for data normalization. Independent studies have proposed other miRNAs as candidate housekeeping [58, 59], but a global consensus is still lacking.

In all assays measuring a relatively large number of miRNAs, global normalization methods can be applied. For amplification-based array data, a commonly used approach is to use a global measure of miRNA expression profiles, such as the mean or the median, as a calibrator. However, the number of detected miRNAs in the circulation with PCR-based methods is usually around 100, likely not large enough to apply a global normalization approach. Moreover, after the discovery phase, a limited number of candidate miRNA markers usually need further validations, both in technical and in independent case series. However, the same data normalization approach will not be applicable in such subsequent validation studies (usually performed with single assays or a custom card containing a limited number of miRNAs). To overcome this problem, Pizzamiglio et al. [60] recently proposed a comprehensive procedure that starting from a data-driven normalization method based on amplification-based array data identifies a small set of miRNAs to be used as reference for data normalization in view of subsequent validation studies. Alternatively, Kroh et al. [32] suggested a miRNA absolute quantification using a standard curve generated with a synthetic miRNA (synthetic oligonucleotides) to carry out by RT-PCR in parallel with biological samples. This approach appears feasible for individual miRNA marker quantification but cannot be used for multiplex miRNA evaluation. The same authors also proposed a normalization strategy based on spiked-in synthetic RNAs. They recommended the use of three* C. elegans* spiked-in control miRNAs during the denaturation of plasma/serum samples to normalize the biological variability that could affect the extraction efficiency. The synthetic exogenous miRNAs could be measured by RT-PCR together with target miRNA and their mean CT values could be used to express the miRNA data as median-normalized CT values [32].

Normalization is a key point also in microarray-based data [61]. Global normalization methods developed for gene expression analysis (e.g., lowess or quantile normalization) are regularly applied to miRNA expression profiling. However, the reduced number of features (compared with the number of genes measured within a gene expression microarray) raises some doubts about the appropriateness of such methods in the context of miRNA profiling, and new strategies have been proposed, such as a modified least-variant set normalization for miRNA microarray [62].

Sequencing-based quantification of miRNAs is a relatively new technology compared to qRT-PCR and microarrays, and there is a minor standardization of the optimal workflow to be used. At present, the standard method to normalize miRNA-seq data is to divide the reads of each miRNA by the total number of reads mapping to the genome or to the known miRNAs. Methods developed for microarray normalization (e.g., lowess or quantile normalization) have also been applied to miRNA-seq data [63].

However, it is noteworthy that all global normalization methods (independently of the platform used) are based on the assumption that the same total amount of miRNAs is expected in all samples and that only a small percentage of miRNAs is differentially expressed, as both up- and downregulated. Such assumptions are often inappropriate for studies aimed to identify cancer-related circulating biomarkers, because in neoplastic patients, as already mentioned, mainly an increase in circulating miRNAs is expected compared with unaffected individuals. This implies that whereas a general upregulation is present in raw data, not only due to technical biases to be removed but also due to the biological phenomenon under investigation, such a trend could disappear after application of classical normalization strategies. Such a problem was clearly demonstrated in the case of an expected general miRNA downregulation as a consequence of inducible deletion of Dicer1 [64]. Consequently, although widely used in the literature, classical normalization methods are not suitable in many situations when analyzing circulating miRNA profiling data. This implies that further research is needed to identify suitable methods, and, in this context, a ratio-based approach has been proposed [65], that is, like using, in turn, all miRNAs as housekeeping. Such an approach has the advantage of limiting* a priori* assumptions; however, high redundancy is generated in the data and it could become not trivial to establish which miRNA has a relevant role and which functions as a calibrator.

### 3.6. Individual Factors

Besides the potential technical biases discussed above, other critical variables that could have deep implications in the accurate interpretation of circulating miRNA biomarker studies are related to the intrinsic interindividual variability and to the influence of disease-independent factors. Facing circulating miRNAs as cancer biomarker molecules, the first source of variability to be considered is related to the tumor itself. In fact, since miRNA expression patterns are extremely specific for individual cancers, miRNA circulating signatures are expected to be extremely specific for different cancer types or molecular subtypes and for distinct tumor features (e.g., hormone responsiveness, oncogene overexpression, etc.). Moreover, recent evidence suggests that individual variability, such as race [66] and gender [67, 68], as well as external factors and life-style, such as drug assumption [69], smoking habits [70], diet [71, 72], physical activity [73], and many other conditions, could contribute to affect miRNA levels in the circulation. Although some of the interindividual variables, such as race, gender, and age, can be easily analyzed and properly considered, there is also a large array of individual and environmental factors that are hardly verifiable and correctly taken into consideration in the evaluation of circulating miRNAs as disease biomarkers. For example, polymorphisms in miRNA chromosome loci have been described as responsible for differential miRNA expression. In particular, copy number variations (CNVs) occurring in coding regions of the genome also contain miRNA genes across all chromosomes [74]. CNVs that cause miRNA deregulation have been shown to play a role in the occurrence of different diseases [75, 76], and it could be plausible to hypothesize that this type of polymorphism contributes to differences in miRNA expression among individuals, probably also influencing the levels of specific circulating miRNAs.

Among the donor-related factors, diet can be considered as a critical potential confounder of miRNA profiling studies. In fact, miRNA expression profiles have been shown to be affected by a large number of dietary constituents such as resveratrol, curcumin, isoflavones, catechins, indoles, and many other compounds including vitamins A and D (reviewed in [77]). It has also been postulated that many of the miRNAs contained in food, once entered in the circulation, would be largely indistinguishable, at sequence and/or function levels, from endogenous miRNAs, and, in addition, they could cause changes in miRNA concentration through homeostatic mechanisms that regulate circulating miRNA-containing vehicles (including lipoprotein particles and exosomes) [72]. The influence of diet as well as that of other external factors such as the physical activity cannot be taken into account during case selection for circulating miRNA studies. Thus, in the evaluation of results, it has to be considered that miRNA levels could represent mainly a summary of individual behavior rather than of what one suffers from. Even though no extensive information is available, it is plausible that also intraindividual variability could affect circulating miRNAs evaluation because miRNA amount may vary in the same individual over time. Notably, pharmacological treatments could have a profound influence on circulating miRNA levels. A very common medication such as aspirin has, for example, been proven to reduce miR-126 levels in the plasma of patients with type 2 diabetes [69]. Importantly, the effects of chemotherapy and, possibly, of different type and/or treatment schedules of anticancer therapies on circulating miRNA levels could have a profound relevance when such molecules are under evaluation as prognostic cancer biomarkers or to monitor tumor progression. However, the effect of pharmacological treatments on circulating miRNA levels points out an important premise for their use as pharmacodynamic markers.

## 4. Conclusions

Circulating miRNAs hold great promise as cancer biomarkers. However, as we have discussed in this review, before translation into clinical practice, all circulating miRNA findings require further steps of validation and a proper standardization of all preanalytical and analytical procedures, in order to control for all potential technical biases. The development and the application of SOPs regulating procedures related to circulating miRNA analysis are in fact an imperative issue to successfully translate miRNA signatures in clinically meaningful tests. Standardized and consistent methods need to be applied at many levels, from whole blood collection to plasma/serum preparation, handling, and banking to RNA extraction and miRNA quantification, in order to keep at minimum the interlaboratory differences that could have high repercussion in miRNA results and also limiting incoherencies among different users. Once consensus procedures have been defined and included in circulating miRNA profiling, it will be possible to accurately interpret and compare different study results and, possibly, to identify those miRNAs acting as novel specific and sensitive cancer biomarkers. However, it is worth keeping in mind that if methodological parameters could be correctly considered in study design, some individual and environmental factors could not be properly evaluated and taken into account during circulating miRNA analysis, thus plausibly influencing circulating miRNA application in clinical practice.
